# Evaluation of a continuing professional development strategy on COVID-19 for 10 000 health workers in Ghana: a two-pronged approach

**DOI:** 10.1186/s12960-023-00804-w

**Published:** 2023-03-06

**Authors:** Roxana Salehi, Stephanie de Young, Augustine Asamoah, Sawdah Esaka Aryee, Raymond Eli, Barbara Couper, Brian Smith, Charity Djokoto, Yaa Nyarko Agyeman, Abdul-Fatawu Suglo Zakaria, Nancy Butt, Amma Boadu, Felix Nyante, Gifty Merdiemah, Joseph Oliver-Commey, Lawrence Ofori-Boadu, Samuel Kaba Akoriyea, Megan Parry, Cindy Fiore, Faustina Okae, Archibald Adams, Hannah Acquah

**Affiliations:** 1 Centre for Global Child Health, The Hospital for Sick Children, Toronto, Canada; 2 Ghana College of Nurses and Midwives, Accra, Ghana; 3 Nursing and Midwifery Council of Ghana, Accra, Ghana; 4 Ghana Health Service, Accra, Ghana; 5 University for Development Studies, Tamale, Ghana; 6 Nurses’ and Midwives’ Training College, Tamale, Ghana; 7 University of Ghana Medical Centre, Accra, Ghana; 8 Ghana Infectious Disease Centre, Accra, Ghana

**Keywords:** Continuing professional development, Evaluation, Low-resource settings, E-learning, Nursing education

## Abstract

**Background:**

COVID-19 has created unprecedented challenges for health systems worldwide. Since the confirmation of the first COVID-19 case in Ghana in March 2020 Ghanian health workers have reported fear, stress, and low perceived preparedness to respond to COVID-19, with those who had not received adequate training at highest risk. Accordingly, the Paediatric Nursing Education Partnership COVID-19 Response project designed, implemented, and evaluated four open-access continuing professional development courses related to the pandemic, delivered through a two-pronged approach: e-learning and in-person.

**Methods:**

This manuscript presents an evaluation of the project's implementation and outcomes using data for a subset of Ghanaian health workers (*n* = 9966) who have taken the courses. Two questions were answered: first, the extent to which the design and implementation of this two-pronged strategy was successful and, second, outcomes associated with strengthening the capacity of health workers to respond to COVID-19. The methodology involved quantitative and qualitative survey data analysis and ongoing stakeholder consultation to interpret the results.

**Results:**

Judged against the success criteria (reach, relevance, and efficiency) the implementation of the strategy was successful. The e-learning component reached 9250 health workers in 6 months. The in-person component took considerably more resources than e-learning but provided hands-on learning to 716 health workers who were more likely to experience barriers to accessing e-learning due to challenges around internet connectivity, or institutional capacity to offer training. After taking the courses, health workers' capacities (addressing misinformation, supporting individuals experiencing effects of the virus, recommending the vaccine, course-specific knowledge, and comfort with e-learning) improved. The effect size, however, varied depending on the course and the variable measured. Overall, participants were satisfied with the courses and found them relevant to their well-being and profession. An area for improvement was refining the content-to-delivery time ratio of the in-person course. Unstable internet connectivity and the high upfront cost of data to access and complete the course online were identified as barriers to e-learning.

**Conclusions:**

A two-pronged delivery approach leveraged distinct strengths of respective e-learning and in-person strategies to contribute to a successful continuing professional development initiative in the context of COVID-19.

## Introduction

COVID-19 has created an unprecedented crisis worldwide, highlighting that a well-trained health workforce who can continuously update their skills in response to new pathogens and emergencies is imperative to a population’s health [1–4]. However, health worker education in sub-Saharan Africa still faces numerous challenges that limit progress toward provision of quality care and attainment of Sustainable Development Goal-3 (Good Health and Well-being). These include shortage of qualified faculty and teaching resources, outdated curricula, and lack of institutional capacity [5–7]. In Ghana, these challenges exist in the larger context of health worker shortage, despite significant gains in the last decade, and the unequitable distribution of this workforce across the country’s different regions [8]. Nurses and midwives remain the primary, and, in underserved areas, sometimes the only health workforce. Therefore, investing in their continuing professional development is crucial, so that they can update and enhance their professional skills and keep themselves and their communities safe [9–11].

Since the confirmation of the first COVID-19 case in Ghana in March 2020 [12], Ghanaian health workers have reported low perceived preparedness to respond to COVID-19. Although several factors contribute to their level of perceived preparedness (e.g., access to personal protective equipment and supportive management), training was found to be the strongest predictor [13]. Health workers who had not received COVID-19-related training were at highest risk for stress, anxiety, and burnout [13, 14]. One of the initiatives that responded to the gap in COVID-19 training to address health worker preparedness was SickKids-Ghana Paediatric Nursing Education Partnership COVID-19 Response Project, hereafter referred to as “the project.” It set out to train 10 000 health workers in Ghana by designing and implementing four relevant continuing professional development courses, provided at no cost to course participants.

The implementing partners for this project include Ghana College of Nurses and Midwives (GCNM), Ghana Health Service (GHS), Ministry of Health (MoH) Ghana, and the Centre for Global Child Health at The Hospital for Sick Children (SickKids), Canada. The project, funded by the Government of Canada through Global Affairs Canada, is an extension of the SickKids-Ghana Paediatric Nursing Education Partnership (PNEP) and builds on 10 years of collaboration between SickKids and its Ghanaian partners [15, 16]. Courses were designed by the project’s content experts in Ghana and Canada, and one of the project’s primary stakeholders, the Nursing and Midwifery Council of Ghana (N&MC) accredited and promoted the courses.

Courses were delivered through a "two-pronged strategy": e-learning and in-person. The courses were also adapted for and made available to health workers outside of Ghana. The project, which continues until April 2023, at the time of writing (July 2022) has surpassed its targets. A total of 18 855 health workers in Ghana and outside of Ghana (primarily from East and West Africa) have completed at least one of the four courses. This manuscript presents the results of evaluating the project's implementation and outcomes using data for a subset of Ghanaian health workers who have taken the courses (*n* = 9966).

## Key evaluation questions

This evaluation answers two questions:

1. How successful was the project in implementing the two-pronged strategy (e-learning and in-person) for the four professional development courses?
2. To what extent did the courses contribute to strengthening health workers' capacity regarding COVID-19 prevention and health promotion?

Implementation success [17–19] was defined by stakeholders as (i) reaching a broad range of frontline health workers across Ghana, including in underserved areas; (ii) delivering courses that are relevant to Ghanaian health workers' learning needs; and (iii) efficient resource use (time, human resources, technology, and financial) to deliver the courses. Health workers' capacity is defined in "Measures" section.

## Existing evaluations: the larger context

Currently, there is a gap in the evaluation of continuing professional development efforts in low-resource settings, especially e-learning interventions [6, 20, 21]. Well-designed e-learning interventions—those delivered through information and communication technology using a variety of instructional designs [6, 20]—can potentially alleviate the burden of faculty shortages and provide access to affordable education irrespective of geography. They offer a convenient way to gain knowledge and skills and are easier to update than curricula delivered by other means [22]. Financially, they cost less than traditional face-to-face training because of reduced costs associated with human resources, travel, and institutional infrastructure, and enable scaling up the reach of training at a comparatively low cost [23, 24]. Many health workers in Ghana already use their mobile phones for learning purposes, have a positive attitude toward e-learning, and exhibit high levels of preparedness for using e-learning interventions [25, 26]. Challenges with internet connectivity and the cost of data are recognized barriers to maximizing the benefits of e-learning interventions, particularly in rural areas [6, 25, 26].

Evaluations comparing the quality of e-learning to traditional in-person learning have produced mixed results. Some argue that e-learning can produce comparable or better gains in knowledge and practice [27]; others suggest that in-person training is superior, because it offers opportunities for hands-on exercises [22]. A meaningful comparison, however, depends on assessing the specific context surrounding the training (course duration, topic complexity, technology used, learners' baseline knowledge, trainers' level of expertise) and its evaluation (design, sample size, types of variables). For example, training on specialized topics, training combined with supportive supervision, and training whose evaluations are conducted right afterward generally have a larger effect size compared to general topic courses, training without supportive supervision, and training with evaluations undertaken months afterward [28, 29]. Finally, effect sizes are generally larger when the training intervention has a targeted sample specifically selected based on the assumption that participants will benefit, rather than when the sample is more diverse [29, 30]. Unfortunately, much of the existing evaluation literature lacks this important contextual information [23, 28, 31, 32].

## Curriculum

From May to August 2021, the Ghana College of Nurses and Midwives and the SickKids Centre for Global Child Health developed the following courses, working in collaboration with experts from the Ghana Health Service and Ministry of Health:

- Course One: COVID-19 in Ghana: Prevention and Health Promotion (e-learning),
- Course Two: COVID-19 Vaccines in Ghana: Communication and Behaviour Change (e-learning),
- Course Three: COVID-19 in Ghana: Child and Adolescent Health (e-learning), and
- Course Four: COVID-19 in Ghana: Promoting Physical and Mental Health of Children, Families, and Health Workers (in-person).

The courses were designed to target a wide range of health workers including nurses, midwives, and physicians. The first three courses were designed for delivery via the World Continuing Education Alliance (WCEA) e-learning platform, while Course Four was designed for in-person delivery. The e-learning courses included narrative descriptions, interactive activities, and case studies where learners applied their knowledge. The participants could take as many of the e-learning courses as they wanted. The in-person course allowed for additional multi-modal teaching and learning strategies, including skills stations, role play, and case presentations. The participants of the in-person course were also provided with technical support to access the e-learning platform and were encouraged to take the other three courses. All four courses included a pre-and post-knowledge test and a commitment to change action plan. In January 2022, courses were updated to reflect new information about COVID-19 and its management.

## Implementation

### Approach 1: e-learning

The e-learning courses were launched in September 2021. The World Continuing Education Alliance (WCEA) e-learning platform allows users to download course content to their smartphones or other portable devices and continue the course offline. However, users must be connected to the internet to verify course completion and submit answers to multiple-choice exams and evaluation questions and to obtain their certificates. To recruit participants, the Ghana College of Nurses and Midwives and the Nursing and Midwifery Council (Ghana) promoted the courses through their websites and social media platforms. WCEA also profiled the courses on their platform to encourage participation.

### Approach 2: in-person

In-person training started in November 2021 while adhering to COVID-19 risk mitigation safety protocols. A targeted approach was used to recruit health workers from identified rural areas with poor internet connectivity and who do not have frequent training opportunities. The Ghana Health Service Director-General, regional and district managers, facility managers, and the project team collaborated to select participants. Similarly, the head offices of the Christian Health Association of Ghana (CHAG) and the Ahmadiyya Muslim Mission nominated participants. During the in-person course, participants not only developed competencies around COVID-19, but they also received support to navigate the WCEA platform and were encouraged to take additional e-learning courses to support their ongoing professional development.

## Methodology

Ethics approval was obtained from the Ghana Health Service Ethics Review Committee in October 2021. A set of evaluation questionnaires were designed by curriculum development team members and the project's evaluation team. A group of graduates from the Ghana College of Nurses and Midwives piloted the evaluation tools to ensure clarity of language. For in-person sessions, questionnaires identical to those used in the e-learning courses were filled out using paper surveys.

### Data included and missing data

The results presented here focus on 9966 health workers who either took the e-learning courses between October 26, 2021, and February 22, 2022 (*n* = 9250), or attended one of 19 two-day in-person courses offered between December 29, 2021, and April 2, 2022 (*n* = 716). The missing data consists of (i) individuals who chose not to answer certain evaluation questions, and (ii) those who did not click on the last button on the e-learning platform and did not go to the page containing the evaluation questions and their certificate of completion. A push notification was sent out to remind and encourage the learners to complete the process, but it did not improve the rates of missing data.

### Measures

Implementation: Reach was captured using a demographic survey completed after each course. A satisfaction survey (5-point scale) with an open-ended text question measured the relevance of the strategy. The strategy’s efficiencies were measured qualitatively by analyzing ongoing partner discussions throughout the project. Although comprehensive economic analysis was outside the scope of the evaluation, the financial cost of developing and delivering the courses was monitored to inform implementation. Cost categories included Human Resources (HR) and meeting costs for curriculum development, HR and platform costs for e-learning, and HR and travel costs for in-person training.

Outcomes: Health workers' capacity measurement was informed by Finn and Colleague's conceptual framework [33] and used five variables:

- Confidence in addressing misinformation about COVID-19 with patients, colleagues, and community members (5-point scale),
- Ability to communicate effectively to support individuals experiencing negative impact of COVID-19 (5-point scale),
- Recommending the COVID-19 vaccine to patients and colleagues, if available (5-point scale),
- Knowledge about COVID-19 using the score of a multiple-choice exam covering key aspects of the curriculum in each course (zero to 100), and
- Comfort level with e-learning (5-point scale).

Responses were captured using pre–post-surveys.

### Analytical approach

Statistical analysis was performed using IBM SPSS Statistics, version 25. Descriptive statistics were conducted to summarize the characteristics of the participants. The distribution of variables was analyzed and, subsequently, Wilcoxon Signed Ranks test for paired samples was used to measure the capacity of the participants regarding COVID-19 prevention and health promotion before and after each course. The only exception was the multiple-choice exam score of Course Four (in-person): due to logistical challenges, it was not possible to link the pre- and post-knowledge scores for this group; therefore, the Mann–Whitney *U* test was used. The significance level was set at 0.01. Effect sizes associated with Wilcoxon Signed Ranks tests and Cohen's criteria of 0.1 small effect, 0.3 medium effect, and 0.5 large effect were used to report findings [35, 36]. Since these thresholds are arbitrary and fail to consider important differences in characteristics of educational intervention, the practical significance of the results was determined by ongoing team and stakeholder discussions and situating results within the relevant literature [29, 30, 34]. A total of 4 034 qualitative comments for the open-ended survey question were analyzed using a thematic content analysis method [35], identifying the top five categories of comments. Percentages in tables are rounded and might not add up to exactly 100.

## Results

### Reach

As shown in Tables 1 and 2, 75% of participants were under the age of 34. Female health workers accounted for 71% of learners. Most of the participants were general nurses, enrolled nurses, or community health nurses. The top three facility types were district hospitals, health centres, and Community-based Health Planning and Services (CHPS) compounds. Participants in e-learning courses followed these overall demographic trends. Participants in the in-person course, however, were 52% female, consisted of 19% midwives (third highest frequency), and the top facility type in which they worked was CHPS compounds.

**Table 1:** Characteristics of 9966 Ghanaian health workers who took the four courses

	Total (*n* = 9966)		E-learning (*n* = 9250)		In-Person (*n* = 716)	
	*n*	%	*n*	%	*n*	%
Age						
< 24	179	1.8	175	1.9	4	0.6
25–34	7286	73.2	6764	73.2	522	72.9
35–44	1871	18.8	1712	18.6	159	22.3
45–54	102	1.1	87	1.0	15	2.1
55 +	43	0.5	40	0.5	3	0.4
Missing	485	4.9	472	5.2	13	1.8
Gender						
Female	7100	71.2	6726	72.7	374	52.2
Male	2805	28.1	2476	26.7	329	45.9
Missing	61	0.6	48	0.5	13	1.8
Primary qualification						
General nurse	3459	34.3	3256	35.2	203	28.4
Enrolled nurse	1696	16.9	1645	17.8	51	7.2
Community health nurse	1220	12.1	1055	11.4	165	23.1
Midwife	1145	11.4	1010	10.9	135	18.9
Public health nurse	248	2.5	178	1.9	70	9.8
Physician or Surgeon	112	1.2	112	1.2	0	0
Other^a^	725	7.2	648	7.0	77	10.8
Missing	1361	13.5	1346	14.4	15	2.1
Level of Facility						
District hospital	3137	31.1	2964	32.1	173	24.2
Health centre	1683	16.7	1488	16.1	195	27.3
CHPS compound	993	9.9	779	8.5	214	29.9
Teaching hospital	754	7.5	754	8.2	0	0
Regional hospital	441	4.4	419	4.6	22	3.1
Poly clinic	439	4.4	421	4.6	18	2.6
Academic institution	108	1.1	108	1.2	0	0
Other^b^	1051	10.5	971	10.5	80	11.2
Missing	1360	13.5	1346	14.6	14	2.0

*CHPS* Community-based Health Planning and Services

^a^For e-learning courses, the 'other' category included paediatric nurses, mental health nurses, administrators and managers, students, and health assistants. For in-person courses, the 'other' category included health promotion officers, disease control officers, health assistants, and mental health nurses

^b^For e-learning courses, the 'other' category includes the Ministry, private facilities, and NGOs. For in-person courses, the 'other' category included Regional Directorate, District Health Directorate, Municipal Hospital, Mission Hospital, and Municipal Health Directorate

**Table 2:** Geographic distribution of 9966 Ghanaian health workers who took the courses

	Total (*n* = 9966)		E-learning (*n* = 9250)		In-Person (*n* = 716)	
	*n*	%	*n*	%	*n*	%
**Region**						
Central	1256	12.7	1256	13.6	0	0
Greater Accra	1215	12.2	1215	13.1	0	0
Eastern	971	9.8	971	10.5	0	0
Ashanti	834	8.4	834	9	0	0
Volta	853	8.6	740	8	113	15.8
Upper West	558	5.6	558	6	0	0
Western	487	4.9	487	5.3	0	0
Northern	388	3.9	299	3.2	89	12.5
Western North	338	3.4	237	2.6	101	14.2
Savannah	272	2.8	223	2.4	49	6.9
Upper East	216	2.2	216	2.3	0	0
Bono	309	3.2	207	2.2	102	14.3
Oti	308	3.1	207	2.2	101	14.2
Bono East	239	2.4	188	2	51	7.2
North East	186	1.9	138	1.5	48	6.8
Ahafo	177	1.8	128	1.4	49	6.9
Missing	1359	13.7	1346	14.6	13	1.9

Table 2 shows that 25% of all participants came from Central and Greater Accra Regions. Less than 4% were from North East and Ahafo regions. While the participants of e-learning courses followed these geographic trends, the participants in the in-person study mainly came from Volta, Western North, Bono, and Oti regions, reflecting the recruitment strategy described in "Approach 2: in-person" section.

### Relevance

Mean overall satisfaction for Courses One (health promotion), Two (vaccine), Three (child and adolescent), and Four (physical and mental health) was 4.49 ± 0.59 (*n* = 6381), 4.49 ± 0.59 (*n* = 3385), 4.47 ± 0.57 (*n* = 2553), and 4.73 ± 0.48 (*n* = 714), respectively. For e-learning courses, participants described the learning as "relevant to my work" and "well-structured" (Table 3). They described the e-learning environment as "logically laid out" and "a faster, safe, and convenient way of learning." Some felt "encouraged to take other courses on the WCEA app" and "regret[ted] not joining the platform earlier." Interactive photos and videos made learning easier, and participants wanted more of them. However, some participants, especially those working in rural areas, reported that limited network coverage and unstable internet created difficulties for fully downloading the course content, especially photos and videos. Some participants commented about the cost of data associated with downloading and uploading content. A suggestion for improvement was to create a space on the courses' platform, where learners could interact.

**Table 3:** Top five categories of comments by health workers who took the courses

Category	Selected quotes
General short positive comments about the course	"*This module met the mental health needs of healthcare workers […]. The best education I've had on COVID-19 so far*."—Nurse, Course One "*Very educative and interactive. I didn't feel bored*, *unlike some other modules which only involve reading and no activitie*s."—Registered community nurse, Course Two "[*It] was impactful, involved presentations from participants and role play which was educative*."—General nurse, Course Four
Knowledge, skills, and confidence gained	*"[…] it was in clear and simple language which will enable me to explain everything about COVID-19 to my community members."*—Enrolled nurse, Course Two *"I had my doubts about the vaccine even as a health worker, but all that is cleared now"*—Registered nurse, Course Two *"The module motivated [me] and increased my skills on management of children and adolescents with COVID-19."*—Midwife, Course Three *"Not so many people are confident taking e-learning courses and exams, including me, but after this module, my confidence is boosted and want to do more courses here."—*Nurse, Course One
Challenges of e-learning	*"It is data consuming to [download]!"*—Enrolled nurse, Course One *"To access this course online is very complicated, especially to those who don't have much knowledge in ICT and [live in] hard-to-reach areas."*—Midwife, Course One *"At times, you will be eager to work, but the network will be disturbing."*—Midwife, Course Four
Opportunities presented by e-learning	*"I am encouraged to take other courses on the WCEA app."*—Midwife, Course One *"It is faster, safe and convenient way of learning and updating oneself on the job content."*—Enrolled nurse, Course Three *"The app is a good one, and I regret not joining earlier."*—Community health nurse, Course Three *"The course was very informative and had awakened my desire to take more online CPD courses."*—General nurse, Course Four
Areas for improvement/suggestions	*“Because the presentations are packed [it] should have been a three day training."*—Registered community nurse, Course Four *"Please, more video demonstration or picture demonstration for further studies."*—Registered community nurse, Course One *"The course is very involving, but the CPD point earned is too small. So if anything can be done about it?"*—Midwife, Course One *"Is it possible, a platform where individuals can interact and share ideas or even study together?"*—General nurse, Course Two *"The training is very useful and should be extended to cover more healthcare workers."*—Public health nurse, Course Four

Participants who completed Course Four (in-person) described it as "helpful" and "the best." They appreciated the opportunity to participate in this course, and comments were made about the need for this type of training to be conducted in person "due to poor network service in rural settings." A frequent comment was the wish for training to be repeated for other health workers "to also build their capacity." Case-based learning was viewed positively, leading to "better understanding of the material." The facilitators and the organization of the course were described as "superb." Areas for improvement included dedicating more time to training, given the content volume (Table 3).

### Efficiency

Decisions regarding resource efficiency (time, human resources, technology, and financial) took place throughout the project, during both curriculum development and implementation. Curriculum development meetings between experts in Ghana and Canada took place virtually. The content team made efforts to avoid duplication of content and to focus on specific priority areas, such as child health and vaccine hesitancy, for which limited open access content was available.

Core curriculum development cost approximately 35% of the total investment for the project between May 2021 and April 2022. E-learning implementation cost 10% of the total investment for the project in the same period and reached 9250 health workers across Ghana. The World Continuing Education Alliance (WCEA) platform was selected, because over 80 000 nurses, midwives, and students in Ghana were already registered on it, representing a broad geographical reach. WCEA’s integration with Nursing and Midwifery Council, Ghana's system for continuing professional development, facilitated course accreditation.

Implementation of the in-person strategy (Course Four) cost 55% of the total investment, reaching 716 health workers living in underserved regions. Regional trainers were chosen to deliver the course, cascading training nationwide and strengthening regional training capacity.

### Participant's capacity

#### Course one: prevention and health promotion

A total of 7243 individuals completed Course One (Table 4). The statistically significant shift in levels of agreement (strongly agree or agree) with the statements ‘I am able to effectively support individuals experiencing negative impact of COVID-19’ increased from 74.7% to 84.9% between baseline and post-training, with a medium effect size. Similarly, the statistically significant shift in levels of agreement in relation to the statement ‘I feel comfortable taking online courses’ increased from 72.2 to 84% with a medium effect size. The shifts in levels of agreement with the statements ‘I feel confident addressing misinformation’ and ‘I would recommend the vaccine’ were statistically significant with small effect sizes. The knowledge score increased by 14% from baseline to after course completion with a medium effect size.

**Table 4:** Participants' capacity before and after taking Course One: Prevention and Health Promotion (*N* = 7243)

Variables^a^	Before		Immediately after		*P*^b^	*Z*^c^	Effect size (*r*)
	*n*	(%)	*n*	(%)			
I feel confident addressing misinformation about COVID-19 with patients, colleagues and community members					< 0.01	− 32.4	− 0.28
*Strongly disagree or disagree*	270	(3.7)	61	(0.8)			
*Neither disagree nor agree*	372	(5.1)	76	(1.0)			
*Agree or strongly agree*	5615	(77.5)	6163	(85.1)			
Missing	986	(13.6)	943	(13.0)			
I am able to communicate effectively to support an individual who is experiencing negative impacts of COVID-19					< 0.01	− 38.5	− 0.34
*Strongly disagree or disagree*	365	(5.0)	52	(0.7)			
*Neither disagree nor agree*	527	(7.3)	110	(1.5)			
*Agree or strongly agree*	5408	(74.7)	6148	(84.9)			
Missing	943	(13.0)	933	(12.9)			
I would recommend the COVID- 19 vaccine to patients and colleagues if it was available					< 0.01	− 31.0	− 0.27
*Strongly disagree or disagree*	212	(2.9)	62	(0.9)			
*Neither disagree nor agree*	365	(5.0)	140	(1.9)			
*Agree or strongly agree*	5727	(79.1)	6114	(84.4)			
Missing	939	(13.0)	927	(12.8)			
I feel comfortable taking an online CPD					< 0.01	− 36.3	− 0.32
*Strongly disagree or disagree*	467	(6.4)	64	(0.9)			
*Neither disagree nor agree*	592	(8.2)	163	(2.3)			
*Agree or strongly agree*	5233	(72.2)	6083	(84.0)			
Missing	951	(13.1)	933	(12.9)			
Knowledge (mean score ± SD) ^d^	74.8 ± 17.0		85.5 ± 14.1		< 0.01	− 46.9	− 0.39

^a^All Likert variables were 5-point scale (1 = strongly disagree, 2 = disagree, 3 = neither disagree nor agree 4 = agree, 5 = strongly agree). Mean score for the Knowledge variable is out of 100

^b^Wilcoxon Signed Ranks Test, 2-tailed for paired samples, was used for all comparisons

^c^Based on negative ranks

^d^(n for pre-knowledge test = 6799, *n* for post-knowledge test = 7243)

#### Course two: vaccine

A total of 4023 individuals completed Course Two (Table 5). The statistically significant shifts in levels of agreement (strongly agree or agree) with the statements ‘I feel confident addressing misinformation’ and ‘I am able to effectively support individuals experiencing negative impact of COVID-19’ increased between baseline and post-training, from 71.7% to 81.1% and from 70.9% to 80.9%, respectively. Both effect sizes were medium. Similarly, the statistically significant shift in levels of agreement in relation to the statement ‘I feel comfortable taking online courses’ increased from 71.6% to 80.6% with a medium effect size. The shifts in levels of agreement with the statement ‘I would recommend the vaccine’ was statistically significant with small effect size. The knowledge score increased by 32% from baseline to after course completion with a medium effect size. The 'clear language' used in this course was considered helpful in transmitting knowledge about COVID-19 to the larger community. Some learners said the course had 'eradicated misconceptions' and 'cleared doubts about the vaccine' (Table 3).

**Table 5:** Participants' capacity before and after taking Course Two: Vaccine (*N* = 4023)

Variables^a^	Before		Immediately after		*P*^b^	*Z*^c^	Effect size (*r*)
	*n*	(%)	*n*	%			
I feel confident addressing misinformation about COVID-19 with patients, colleagues and community members					< 0.01	− 24.9	− 0.31
*Strongly disagree or disagree*	201	(5.0)	31	(0.8)			
*Neither disagree nor agree*	217	(5.4)	46	(1.1)			
*Agree or strongly agree*	2883	(71.7)	3262	(81.1)			
Missing	722	(17.9)	684	(17.0)			
I am able to communicate effectively to support an individual who is experiencing negative impacts of COVID-19					< 0.01	− 27.5	− 0.33
*Strongly disagree or disagree*	205	(5.1)	20	(0.5)			
*Neither disagree nor agree*	286	(7.1)	73	(1.8)			
*Agree or strongly agree*	2854	(70.9)	3256	(80.9)			
Missing	678	(16.9)	674	(16.8)			
I would recommend the COVID-19 vaccine to patients and colleagues if it was available					< 0.01	− 24.3	− 0.29
*Strongly disagree or disagree*	102	(2.5)	23	(0.6)			
*Neither disagree nor agree*	213	(5.3)	68	(1.7)			
*Agree or strongly agree*	3027	(75.2)	3249	(80.8)			
Missing	681	(16.9)	683	(17.0)			
I feel comfortable taking an online CPD					< 0.01	− 25.5	− 0.31
*Strongly disagree or disagree*	203	(5.0)	27	(0.7)			
*Neither disagree nor agree*	251	(6.2)	70	(1.7)			
*Agree or strongly agree*	2879	(71.6)	3242	(80.6)			
Missing	690	(17.2)	684	(17.0)			
Knowledge (mean score ± (SD)) ^d^	62.3 ± 17.8		82.0 ± 16.1		< 0.01	− 43.9	− 0.37

^a^All Likert variables were 5-point scale (1 = strongly disagree, 2 = disagree, 3 = neither disagree nor agree 4 = agree, 5 = strongly agree). Mean score for the Knowledge variable is out of 100.

^b^Wilcoxon Signed Ranks Test, 2-tailed for paired samples, was used for all comparisons

^c^Based on negative ranks

^d^(*n* for pre-knowledge test = 3333, *n* for post-knowledge test = 3339)

#### Course three: child and adolescent health

A total of 2892 individuals completed Course Three (Table 6). The statistically significant shift in levels of agreement (strongly agree or agree) with the statements ‘I am able to effectively support individuals experiencing negative impact of COVID-19’ increased between baseline and post-training, from 76.6% to 85%. Similarly, the statistically significant shift in levels of agreement in relation to the statement ‘I feel comfortable taking online courses’ increased from 75.1% to 84.5%. Both effect sizes were medium. The shifts in levels of agreement with the statement ‘I feel confident addressing misinformation’ and ‘I would recommend the vaccine’ were statistically significant with small effect sizes. The knowledge score increased by 25% from baseline to after course completion with a medium effect size.

**Table 6:** Participants' capacity before and after taking Course Three: Child and Adolescent Health (*N* = 2892)

Variables^a^	Before		Immediately after		*P*^b^	*Z*^c^	Effect size (*r*)
	*n*	%	*n*	%			
I feel confident addressing misinformation about COVID-19 with patients, colleagues and community members					< 0.01	− 20.4	− 0.28
*Strongly disagree or disagree*	122	(4.2)	21	(0.7)			
*Neither disagree nor agree*	155	(5.4)	40	(1.4)			
*Agree or strongly agree*	2218	(76.7)	2457	(85)			
Missing	397	(13.7)	374	(12.9)			
I am able to communicate effectively to support an individual who is experiencing negative impacts of COVID-19					< 0.01	− 22.3	− 0.31
*Strongly disagree or disagree*	128	(4.4)	22	(0.8)			
*Neither disagree nor agree*	176	(6.1)	39	(1.3)			
*Agree or strongly agree*	2214	(76.6)	2457	(85.0)			
Missing	374	(12.9)	374	(12.9)			
I would recommend the COVID-19 vaccine to patients and colleagues if it was available					< 0.01	− 20.8	− 0.29
*Strongly disagree or disagree*	73	(2.5)	15	(0.5)			
*Neither disagree nor agree*	138	(4.8)	61	(2.1)			
*Agree or strongly agree*	2303	(79.6)	2433	(84.1)			
Missing	378	(13.1)	383	(13.2)			
I feel comfortable taking an online CPD					< 0.01	− 21.7	− 0.31
*Strongly disagree or disagree*	142	(4.9)	21	(0.7)			
*Neither disagree nor agree*	188	(6.5)	48	(1.7)			
*Agree or strongly agree*	2172	(75.1)	2445	(84.5)			
Missing	390	(13.5)	378	(13.1)			
Knowledge (mean score ± (SD)) ^d^	69.8 ± 17.6		87.3 ± 15.2		< 0.01	− 36.1	− 0.37

^a^All Likert variables were 5-point scale (1 = strongly disagree, 2 = disagree, 3 = neither disagree nor agree 4 = agree, 5 = strongly agree). Mean score for the Knowledge variable is out of 100

^b^Wilcoxon Signed Ranks Test, 2-tailed for paired samples, was used for all comparisons

^c^Based on negative ranks

^d^(*n* for pre-knowledge test = 2694, *n* for post-knowledge test = 2892

#### Course four: mental and physical health

A total of 716 individuals completed Course Four (Table 7). The statistically significant shifts in levels of agreement (strongly agree or agree) with the statements ‘I feel confident addressing misinformation’ and ‘I feel comfortable taking online courses’’ increased between baseline and post-training, from 79.1% to 99.3% and from 66.6% to 98.2%, respectively. Both effect sizes were large. The shifts in levels of agreement with the statements ‘I am able to effectively support individuals experiencing negative impact of COVID-19’ and ‘I would recommend the vaccine’ were statistically significant with medium effect sizes. The knowledge score increased by 19% from baseline to after course completion with a medium effect size. Participants reported being encouraged to take online courses (Table 3).

**Table 7:** Participants' capacity before and after taking Course Four: Mental and Physical Health (*n* = 716)

Variables ^a^	Before		Immediately after		*P*^b^	*Z*^c^	Effect size (*r*)
	*n*	%	*n*	%			
I feel confident addressing misinformation about COVID-19 with patients, colleagues and community members					< 0.01	− 20.4	− 0.53
*Strongly disagree or disagree*	69	(9.6)	0	(0.0)			
*Neither disagree nor agree*	75	(10.5)	0	(0.0)			
*Agree or strongly agree*	566	(79.1)	711	(99.3)			
Missing	6	(0.8)	5	(0.7)			
I am able to communicate effectively to support an individual who is experiencing negative impacts of COVID-19					< 0.01	− 22.3	− 0.33
*Strongly disagree or disagree*	47	(6.6)	2	(0.3)			
*Neither disagree nor agree*	61	(8.5)	1	(0.1)			
*Agree or strongly agree*	603	(84.2)	707	(98.7)			
Missing	5	(0.7)	6	(0.8)			
I would recommend the COVID- 19 vaccine to patients and colleagues if it was available					< 0.01	− 20.8	− 0.45
*Strongly disagree or disagree*	29	(4.1)	3	(0.4)			
*Neither disagree nor agree*	52	(7.3)	2	(0.3)			
*Agree or strongly agree*	629	(87.8)	706	(98.6)			
Missing	6	(0.8)	5	(0.7)			
I feel comfortable taking an online CPD					< 0.01	− 21.7	− 0.38
*Strongly disagree or disagree*	105	(14.7)	1	(0.1)			
*Neither disagree nor agree*	129	(18.0)	4	(0.6)			
*Agree or strongly agree*	477	(66.6)	703	(98.2)			
Missing	5	(0.7)	8	(1.1)			
Knowledge (mean score ± SD) ^d^	66.4 ± 15.8		78.9 ± 15.7		< 0.01	− 36.1	− 0.51

^a^All Likert variables were 5-point scale (1 = strongly disagree, 2 = disagree, 3 = neither disagree nor agree 4 = agree, 5 = strongly agree). Mean score for the Knowledge variable is out of 100

^b^Wilcoxon Signed Ranks Test, 2-tailed for paired samples, was used for all comparisons except for comparing the knowledge score for which a Mann–Whitney *U*-test was used

^c^Based on negative ranks

^d^(*n* for pre-knowledge test = 716, *n* for post-knowledge test = 716)

## Discussion

Judged against the evaluation criteria (broad reach, efficiency, and relevance) the implementation of the strategy was found to be successful. Rapid curriculum development using virtual meetings was made possible by a well-established partnership, and good working relationships between Ghanaian and Canadian partners. Investment was maximized by delivering training to a broad range of health workers across Ghana using a two-pronged strategy of e-learning and in-person training, each offering benefits and challenges in a Ghanaian, and pandemic, context.

The speed at which health workforce training can be scaled up is particularly important during a pandemic, when timely information is essential. Despite some initial concerns about the feasibility of this approach, particularly around the reach and uptake of e-learning courses, this strategy was successfully implemented, reaching a large number of health workers (*n* = 9250) across Ghana in a 6-month period. Awareness building of the courses and choosing a learning platform that was already known to health workers in Ghana improved efficiency. However, as has been observed by others [3, 21, 26], the evaluation identified challenges with e-learning, including unstable internet and the cost of data for downloading and uploading content.

As others have observed [25], the in-person training took considerably more resources than e-learning, especially given the need for pandemic-related safety and logistics. Compared to e-learning, fewer individuals (*n* = 716) were able to be trained via this training modality. However, the in-person course offered hands-on learning to health workers who needed COVID-19-related training and were more likely to experience barriers to accessing e-learning.

Learners found the courses relevant to their needs and their feedback identified several implication for future design of such courses. For the e-learning courses, interactive content, visual aid (photos, videos), and small file sizes to help with the download of course content were important. For the in-person course, ensuring the optimal content-to-delivery time ratio and using plain language learning material proved important.

All four courses contributed to strengthening health workers' capacity regarding COVID-19 prevention and health promotion but the effect size (i.e., the amount of change) varied from small to large depending on the course and variable measured (Tables 4, 5, 6 and 7). For e-learning courses, the effect sizes varied from small to medium, and for the in-person course, the effect sizes varied from medium to large. While these effect sizes are useful in painting an overall picture, with education evaluation, a 'small' effect size on a difficult-to-change variable (e.g., attitude toward recommended the vaccine) could be as valuable as a larger effect size on something easier to change (e.g., knowledge) [29–31]. In some situations, the initial levels of confidence were already high at baseline, thus a large change (large effect size) would not be expected or possible.

Although the in-person course is not directly comparable to the e-learning courses due to several key differences (length, teaching methods, and participants' characteristics as described in "Approach 2: in-person" section), the observed large effect sizes for the in-person course were encouraging, albeit expected. As mentioned earlier, the project made concerted efforts to target participants with less access to training opportunities for the in-person course ("Approach 2: in-person" and "Reach" section); targeted samples typically result in larger effect sizes [29]. Furthermore, the in-person course allowed the participants to ask the trainers questions in real-time, work through a hands-on exercise about addressing misinformation, and receive in-person support for accessing the e-platform, which are likely to have contributed to the observed larger effect sizes for these variables (Table 7).

## Limitations and strength

This evaluation lacks a longitudinal perspective as no follow-up evaluations were conducted to determine whether participants were applying what they learned in their practices [36]. It was beyond the scope of the evaluation to conduct a comprehensive economic analysis, and as such, only costs to the program were calculated. Other costs such as the cost to the health system when health workers leave work to participate in training, or the cost of data for e-learners were not analyzed. Similarly, benefits of training master trainers for the in-person course were not part of this cost analysis. Evaluation was done internally by the research teams at SickKids and the Ghana College of Nurses and Midwives, with the potentially biases associated with internal evaluation. Strengths associated with this evaluation include the use of both quantitative and qualitative data [37]. In addition, a broad range of stakeholders were involved in the interpretation of results, making them valid and useful [38]. Finally, this evaluation contributes to bridging a gap in the assessment of large-scale continuing professional development efforts in low-resource settings, especially e-learning interventions.

## Conclusions

The success of the strategy was due to well-established partnerships, the quality and relevance of the curriculum, and the two-pronged delivery approach which maximized reach while reducing barriers to accessing education. Health workforce training efforts need to be accompanied by other investments in health systems, notably facility infrastructure, faculty development, and good quality data.

## Data Availability

The data sets supporting the conclusions of this article are available on request from the corresponding author.

## Abbreviations

CPD: Continuing professional development
GHS: Ghana Health Service
GCNM: Ghana College of Nurses and Midwives
MoH: Ministry of Health
N&MC: Nursing and Midwifery Council of Ghana
WCEA: World Continuing Education Alliance
PNEP: SickKids-Ghana Paediatric Nursing Education Partnership
